# Cardiorenal and Metabolic Convergence in Acute Heart Failure: Severe Cardiorenometabolic Syndrome as a High-Risk Phenotype

**DOI:** 10.3390/biomedicines14020467

**Published:** 2026-02-20

**Authors:** Raquel López-Vilella, Borja Guerrero Cervera, Julia Martínez Solé, Sara Huélamo Montoro, Víctor Donoso Trenado, Mireia Company Langa, Valero Soriano Alfonso, Luis Martínez Dolz, Luis Almenar-Bonet

**Affiliations:** 1 Heart Failure and Transplantation Unit, Hospital Universitari i Politècnic La Fe, 46026 Valencia, Spain; cune10@hotmail.com (R.L.-V.); juliamsole@gmail.com (J.M.S.); vdonoso@outlook.com (V.D.T.);; 2 Advanced Cardiorenal Unit, Hospital Universitari i Politècnic La Fe, 46026 Valencia, Spain; 3 Cardiology Department, Hospital Universitari i Politècnic La Fe, 46026 Valencia, Spain; sarahlm395@gmail.com (S.H.M.); mireiacompanylanga@gmail.com (M.C.L.); valero1996@gmail.com (V.S.A.);; 4 Centro de Investigación Biomédica en Red de Enfermedades Cardiovasculares (CIBERCV), Instituto de Salud Carlos III, 28029 Madrid, Spain

**Keywords:** acute heart failure, cardiorenometabolic syndrome, cardiorenal syndrome, prognosis

## Abstract

**Background:** Cardiorenometabolic syndrome (CRMS) reflects the interaction between heart failure (HF), chronic kidney disease, and metabolic disorders. Its prognostic impact during the acute phase of HF remains poorly defined. The primary objective of this study was to assess whether severe CRMS (sCRMS: estimated glomerular filtration rate <45 mL/min/1.73 m^2^ associated with type 2 diabetes mellitus and/or obesity) predicts worse clinical outcomes. **Methods:** This was a retrospective observational study of a prospective cohort including 2228 patients admitted for acute HF between 2015 and 2025. Clinical characteristics and outcomes (mortality, HF readmission, and the composite endpoint) were compared between patients with and without sCRMS. **Results:** sCRMS was present in 486 patients (21.8%) who were older, had worse functional class, and a higher burden of cardiovascular comorbidities. They presented more frequently with systemic congestion and less often with de novo HF. During follow-up, sCRMS was associated with higher mortality (29.4% vs. 18.4%), HF readmissions (56.2% vs. 33.5%), and the composite endpoint (85.6% vs. 51.9%) (all *p* < 0.001). In multivariable analysis, sCRMS remained an independent predictor of mortality (HR 1.25), readmissions (HR 1.24), and overall morbidity and mortality (HR 1.20). **Conclusions:** In patients hospitalized for acute HF, sCRMS consistently identified a clinically vulnerable phenotype with an unfavorable prognosis. These findings support the value of sCRMS as a simple and reproducible prognostic marker and highlight the need for integrated cardiorenometabolic strategies during post-discharge follow-up.

## 1. Introduction

Cardiorenometabolic syndrome (CRMS) describes the bidirectional and self-perpetuating interaction among the cardiovascular, renal, and metabolic systems. In 2023, the American Heart Association (AHA) unified this concept under the term Cardiovascular–Kidney–Metabolic (CKM) syndrome, established staging criteria, and emphasized that the coexistence of heart failure (HF), chronic kidney disease (CKD), and metabolic factors (type 2 diabetes mellitus [T2DM] and obesity) markedly increases mortality and hospital readmissions [1,2]. Recent reviews confirm that these three axes share inflammatory, neurohormonal, and oxidative stress pathways, which explains how frequently they coexist in the same patient [2].

Acute cardiorenal syndrome (CRS type 1), defined as acute kidney injury secondary to cardiac decompensation, is common (≈30–50%) among patients hospitalized for acute HF and is associated with a worse prognosis [3]. In the setting of acute HF, convergence of CRMS is not uncommon: contemporary registries report CKD in approximately 40% of patients and T2DM or obesity in more than 50% of admissions [4,5], with a negative impact on survival and quality of life. In this scenario, contemporary diagnostic strategies emphasize early clinical assessment of congestion, integration of biomarkers such as natriuretic peptides, and evaluation of renal function to guide risk stratification and management. Current therapeutic approaches during hospitalization focus on prompt decongestion with intravenous diuretics, careful optimization of hemodynamics, and early initiation or continuation of guideline-directed medical therapy when feasible. Recent guidelines also highlight the importance of early identification of clinically vulnerable phenotypes to inform treatment intensity and post-discharge planning, particularly in patients with cardiorenal and metabolic comorbidities [6,7].

However, despite the growing relevance of this integrated view in prevention and outpatient management, important knowledge gaps remain in the acute phase of HF, and most CRMS series derive from outpatient or CKD cohorts. There is a lack of analyses specifically focused on the in-hospital phase, where pathophysiology and therapeutic opportunities differ. Moreover, most studies continue to evaluate renal dysfunction and metabolic factors in isolation, without assessing their combined effect, and with heterogeneity in the definition of CRMS.

The study hypothesis was that, in patients with acute HF, the severe CRMS phenotype (sCRMS) would be associated with a higher frequency of major outcomes (mortality and readmissions) compared with patients without this combined metabolic and renal profile, and that specific clinical characteristics might allow risk stratification and help guide future therapeutic interventions during follow-up.

The primary objective of the study was to assess whether the sCRMS phenotype (glomerular filtration rate [GFR] <45 mL/min/1.73 m^2^ plus T2DM or obesity) predicts worse clinical outcomes (mortality or HF readmission) in a large cohort of patients with acute HF. Secondary objectives were to determine the prevalence of sCRMS in acute HF, compare clinical, laboratory, and therapeutic characteristics between patients with and without this phenotype, and analyze predictors of morbidity and mortality.

## 2. Materials and Methods

This was a retrospective study based on a database of patients consecutively admitted for an episode of acute HF to the Cardiology Department of a tertiary care hospital. Data were collected prospectively during hospitalization and subsequently extracted and curated by a team of cardiologists specialized in HF. Recruitment was consecutive over a 10-year period (July 2015–July 2025), with a total of 3406 episodes of acute HF recorded. Elective admissions for planned procedures, transfers from other hospitals, and episodes with insufficient data to calculate estimated glomerular filtration rate (eGFR), ascertain the presence of T2DM, or determine body mass index (BMI) were excluded.

A total of 2228 patients were included in the analysis: 486 with sCRMS and 1742 without this syndrome.

For the present analysis, the sCRMS phenotype was defined as an eGFR <45 mL/min/1.73 m^2^ at admission together with the presence of T2DM or a diagnosis of obesity (BMI ≥30 kg/m^2^ or documented in the medical record).

The diagnosis of acute HF was established according to European guideline criteria, based on the presence of signs and/or symptoms of congestion with objective evidence of cardiac dysfunction and the need for intravenous therapy [8].

Variables of interest included clinical, laboratory, and therapeutic data at admission. The main outcome measures were mortality, readmission, and overall morbidity and mortality during follow-up. Patients were censored at death for the readmission analysis.

### Statistical Analysis

Categorical variables are expressed as percentages, and continuous variables as mean (standard deviation) or median (interquartile range), depending on whether they followed a normal distribution, as assessed by the Kolmogorov–Smirnov test. Group comparisons were performed using the chi-square test with Yates’ correction when appropriate for categorical variables, and Student’s *t* test or the Mann–Whitney *U* test for continuous variables with non-normal distribution.

Survival was analyzed using Kaplan–Meier curves, with comparisons performed using the log-rank test. Multivariable survival analysis was conducted using Cox proportional hazards regression, with the sCRMS variable entered as a dichotomous factor. A forward conditional method was used for variable selection. Variables included were those showing statistical significance in univariable analysis or considered clinically relevant. A *p* value <0.05 was considered statistically significant.

Statistical analyses were performed using IBM SPSS Statistics version 27^®^ and Stata^®^ Statistics/Data Analysis version 16.1, Spain. Figures were generated using SPSS and subsequently edited with PowerPoint 2021 (Microsoft Office).

## 3. Results

### 3.1. Medical History and Clinical Profile

Patients with sCRMS were significantly older, with no relevant differences observed in sex distribution. Regarding underlying heart disease, sCRMS was associated with a higher prevalence of ischemic heart disease and a greater overall burden of cardiovascular comorbidities. In contrast, de novo HF was more frequent among patients without sCRMS.

With respect to prior HF history, patients with sCRMS had a higher number of previous hospitalizations, worse baseline functional class, and a slightly longer length of hospital stay. In addition, the hemodynamic presentation pattern differed between groups: patients with sCRMS more frequently exhibited systemic or mixed congestion, whereas isolated pulmonary congestion predominated in patients without sCRMS (Table 1).

### 3.2. Prior Treatment and Admission Laboratory Findings

Analysis of prior treatment revealed relevant differences between groups. Although no significant differences were observed in the use of angiotensin-converting enzyme inhibitors (ACEIs), angiotensin II receptor blockers (ARBs), angiotensin receptor–neprilysin inhibitors (ARNIs), or mineralocorticoid receptor antagonists (MRAs), patients with sCRMS more frequently received beta-blockers. Consistently, diuretic use was significantly higher in the sCRMS group, including loop diuretics and other strategies for intensification of diuretic therapy. In addition, these patients showed greater use of antiplatelet agents, nitrates, statins, and calcium channel blockers.

In the metabolic and renal domains, patients with sCRMS had higher use of oral antidiabetic agents, sodium–glucose cotransporter 2 inhibitors (SGLT2i), potassium supplements, hypokalemia-inducing agents, and allopurinol (Table 2).

At the laboratory level, patients with sCRMS had significantly higher values of urea, uric acid, N-terminal pro–B-type natriuretic peptide (NT-proBNP), and high-sensitivity troponin T. On complete blood count, lower hemoglobin and hematocrit levels were observed, with a higher prevalence of anemia. In contrast, no significant differences were found in certain renal and congestion biomarkers, such as carbohydrate antigen 125 (CA-125), cystatin C, or microalbuminuria (Table 3).

### 3.3. Diagnostic Criteria and Morbidity–Mortality Data

Analysis of diagnostic criteria confirmed that sCRMS is inherently associated with a higher burden of metabolic comorbidities, with a markedly higher prevalence of T2DM and obesity. From a renal function perspective, patients with sCRMS had significantly higher creatinine levels and clearly lower eGFR, with all patients in this group having an eGFR <45 mL/min/1.73 m^2^.

**Figure 1:**
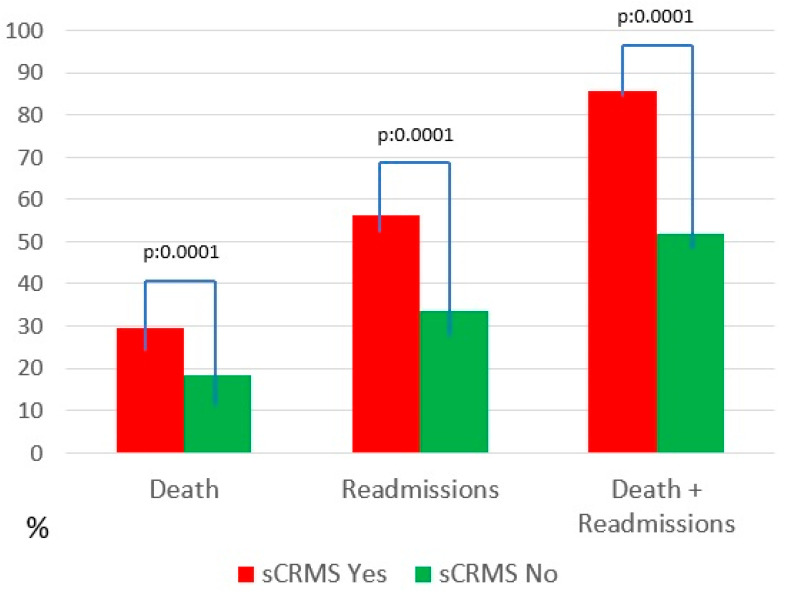
Mortality and readmissions. Abbreviations: sCRMS: Severe cardiorenometabolic syndrome.

Regarding clinical events during follow-up, HF-related mortality, HF readmissions, and—most notably—the combined endpoint of mortality or readmission were significantly more frequent in the sCRMS group (Table 4 and Figure 1). Mortality at 10 years was 29.4% in the severe CRMS group, which was comparable to that observed in patients with isolated renal dysfunction (SCR; 30.2%; *p* = 0.772), and significantly higher than in patients with diabetes alone (24.4%; *p* = 0.037) or obesity alone (11.8%; *p* < 0.001). Regarding heart failure readmissions, severe CRMS was associated with the highest cumulative incidence at 10 years (56.2%), compared with isolated renal dysfunction (51.4%; *p* = 0.092), diabetes alone (44.3%; *p* < 0.001), and obesity alone (42.6%; *p* < 0.001). These findings indicate that while mortality risk was largely driven by renal dysfunction, the coexistence of renal and metabolic disease was associated with a greater burden of recurrent heart failure hospitalizations. Comparative analyses of 10-year outcomes across phenotypes are summarized in Table 5.

### 3.4. Survival Curves and Multivariable Analysis

Survival analysis confirmed the adverse prognostic impact of sCRMS. In the unadjusted analysis, sCRMS was associated with a consistently higher burden of morbidity and mortality, HF readmissions, and the combined endpoint. After multivariable adjustment for relevant clinical variables, these associations were attenuated but remained statistically significant (Table 6).

**Figure 2:**
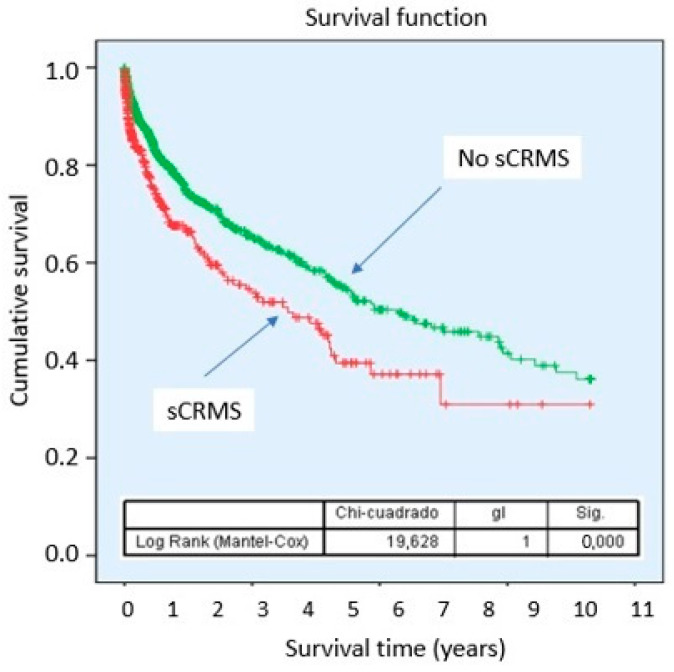
Survival probability of sCRMS. Abbreviations: sCRMS: Severe cardiorenometabolic syndrome.

**Figure 3:**
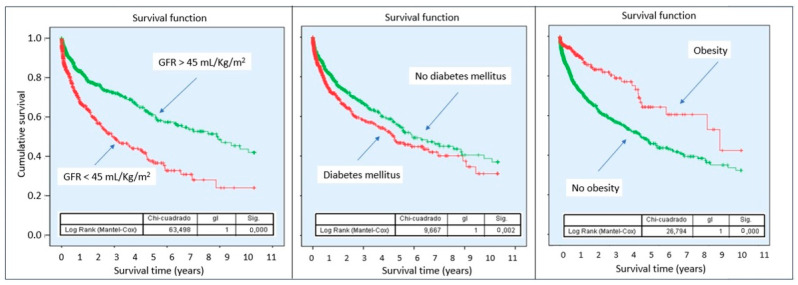
Survival probability according to sCRMS component. Abbreviations: sCRMS: Severe cardiorenometabolic syndrome. Left: Glomerular filtration rate < 45 mL/min/1.73 m^2^; Center: Diabetes mellitus; Right: Obesity.

Kaplan–Meier curves demonstrated a lower cumulative probability of survival in patients with sCRMS throughout follow-up, with progressively diverging curves (Figure 2). Stratified analysis according to the individual components of sCRMS revealed that certain components of the syndrome contribute differentially to mortality risk. Notably, patients with obesity showed a better prognosis compared with those of normal weight (Figure 3).

## 4. Discussion

Acute HF represents a clinical scenario in which hemodynamic decompensation and congestion are closely associated with multiorgan dysfunction, particularly at the renal and metabolic levels. In this context, the coexistence of CKD, T2DM, and obesity has been associated with greater clinical vulnerability and a higher rate of subsequent events during follow-up [1,2]. Despite increasing recognition, evidence on CRMS phenotypes has been generated predominantly in stable or outpatient populations, leaving a knowledge gap in the hospitalized acute HF setting [9]. In this study, the prevalence of sCRMS in this context was 22%, corresponding to a patient profile with a higher burden of cardiovascular comorbidity, worse functional class, and a greater history of prior hospitalizations. Even the pattern of acute decompensation differed, with a higher prevalence of systemic congestion in these patients.

Furthermore, it was observed that patients with sCRMS experienced greater HF-related morbidity and mortality (readmissions and HF-related death) during follow-up, and that the presence of sCRMS at admission was an independent predictor of HF-related morbidity and mortality.

Patients with sCRMS were older, had a higher prevalence of ischemic heart disease, and carried a greater overall burden of cardiovascular comorbidity. Conversely, de novo HF was less frequent in this group, suggesting distinct clinical profiles between the two cohorts. Indeed, patients with sCRMS had a higher number of previous hospitalizations and worse baseline functional class. These findings are consistent with the literature, which reports that, in the context of acute HF, the accumulation of renal and metabolic comorbidities typically identifies older patients with a higher atherosclerotic burden, supporting the notion that sCRMS represents a more “advanced” disease phenotype rather than a single, isolated episode [10,11,12].

From a pathophysiological standpoint, this pattern aligns with cardiorenal syndrome, where renal dysfunction participates in a vicious cycle of congestion, neurohormonal activation, and disease progression [13]. Beyond these clinical differences, the hemodynamic presentation pattern differed between groups: patients with sCRMS more frequently exhibited systemic or mixed congestion, whereas isolated pulmonary congestion predominated in patients without sCRMS. This observation has been described as being closely related to venous congestion and elevated right-sided pressures (right ventricular dysfunction/venous hypertension), which is associated with worse renal function and greater need for combined diuretic therapy [14,15,16]. In line with this, patients with sCRMS had higher use of loop diuretics and diuretic intensification strategies, reflecting the predominance of systemic congestion, which may reflect impaired diuretic responsiveness—a situation particularly common when renal dysfunction coexists with HF [17]. Beyond diuretic therapy, in general, patients with sCRMS had greater exposure to treatments targeting HF and cardiovascular, metabolic, and renal comorbidities. Emerging evidence indicates that pharmacological therapies initially developed for metabolic control also provide substantial cardiovascular and renal benefits; in fact, SGLT2 inhibitors and glucagon-like peptide-1 receptor agonists (GLP-1RA) have shown favorable effects on major cardiovascular outcomes [18]. This therapeutic pattern reflects greater complexity in management, consistent with a more advanced stage of HF and the presence of cardiorenal syndrome [19,20].

When analyzing laboratory findings, we observed that sCRMS is associated with a profile indicative of greater clinical severity, characterized by worse renal function, higher neurohormonal activation, increased myocardial injury, electrolyte disturbances, and anemia. This supports its potential clinical relevance as a marker of risk and clinical complexity in patients hospitalized for HF [13,21]. Notably, NT-proBNP levels were higher in the sCRMS group, which may reflect greater overall disease burden and a higher degree of systemic congestion, a pattern particularly described in the literature among patients with impaired renal function [22,23]. However, no differences were found between groups in CA-125 levels, despite the predominance of systemic congestion in patients with sCRMS. These findings have been previously reported by our group, showing that approximately 25% of patients with acute HF and marked systemic congestion present normal CA-125 levels—especially women, patients with preserved ejection fraction, and those with >50% inspiratory collapse of the inferior vena cava [24,25].

Based on these biomarker findings, several research groups have advocated for the implementation of an integrated cardiorenometabolic laboratory profile to optimize clinical care, reduce healthcare costs, and improve patient outcomes [26,27,28].

Regarding clinical outcomes, patients with sCRMS showed a markedly higher incidence of mortality, HF readmissions, and the combined endpoint. Several studies have demonstrated that the coexistence of T2DM in the setting of acute HF is associated with higher in-hospital mortality and a higher-risk profile, likely mediated by a greater burden of ischemic heart disease and associated comorbidities. Likewise, the development or coexistence of cardiorenal syndrome during episodes of decompensated HF has been robustly linked to worse clinical outcomes, including mortality and early events, becoming established as a marker of systemic vulnerability and severity of the acute episode. Our results indicate that renal dysfunction remains the predominant determinant of long-term mortality in patients hospitalized for acute heart failure, as mortality rates in severe CRMS were comparable to those observed in patients with isolated renal impairment, highlighting the central role of kidney disease within the cardiorenometabolic continuum. Notably, however, patients with severe CRMS experienced the highest rates of heart failure readmissions during follow-up [29,30]. Taken together, these findings support severe CRMS as a pragmatic, admission-based syndromic phenotype reflecting cardiorenometabolic complexity and systemic vulnerability in acute heart failure. This construct is particularly relevant for identifying patients at increased risk of recurrent decompensations and repeated hospitalizations, rather than for outperforming isolated renal dysfunction in mortality prediction.

Survival analysis additionally demonstrated a progressive divergence of the curves over time, suggesting that the prognostic impact of sCRMS is sustained and extends beyond the acute phase of hospitalization. These findings have been reported in the literature, where patients with sCRMS not only experience higher in-hospital mortality but also worse long-term outcomes [31,32]. Importantly, follow-up duration was comparable between groups, supporting the presence of a consistently higher risk profile [33]. In multivariable analysis, sCRMS remained an independent predictor of mortality and HF readmissions, reinforcing its prognostic value beyond traditional clinical risk determinants such as age, baseline functional class, or presentation as de novo HF, although with a modest effect size.

Finally, stratified analysis by individual components of the syndrome revealed relevant prognostic heterogeneity, with a comparatively more favorable prognosis observed among patients with isolated obesity. This finding is consistent with prior descriptions of the so-called “obesity paradox” in heart failure populations, in which adiposity does not uniformly translate into increased risk and may even be associated with better outcomes in certain clinical contexts [34,35,36]. These observations suggest that the relationship between metabolic factors and prognosis in acute HF is unlikely to be strictly linear and may reflect complex interactions involving frailty, body composition, inflammatory status, and disease stage [37].

Overall, these results support the usefulness of integrating renal and metabolic variables into prognostic stratification in acute HF, particularly in a context in which classical models may not fully capture the complexity in patients with cardiorenometabolic multimorbidity [29,34].

From a clinical perspective, early identification of severe CRMS at hospital admission may have relevant practical implications. Although this study does not establish causality or evaluate specific treatment strategies, recognizing this phenotype may support intensified monitoring, careful decongestion, and early optimization of guideline-directed medical therapy. Therapies with proven cardiorenal and metabolic benefits, such as sodium–glucose cotransporter 2 inhibitors, may be particularly relevant in this population. In addition, the high burden of recurrent hospitalizations suggests that structured multidisciplinary management and closer post-discharge follow-up may be warranted. The novelty of this study lies in providing a pragmatic, admission-based definition of severe CRMS tailored to the acute heart failure setting. Rather than proposing a new pathophysiological framework, this approach extends existing cardiorenometabolic models by enabling early phenotypic identification during the acute phase and by capturing differences in clinical trajectory and risk of recurrent decompensation.

This study has several limitations that should be considered when interpreting the results. First, its retrospective observational design precludes the establishment of causal relationships and does not exclude the influence of residual confounding from unmeasured variables. Although multivariable analyses adjusted for several key indicators of heart failure severity and clinical complexity, the observed associations should be interpreted as descriptive and prognostic rather than causal. Potential collinearity among variables related to heart failure severity represents an additional limitation of this study. Parameters such as eGFR, NT-proBNP, anemia, and diuretic use may reflect overlapping aspects of congestion, renal dysfunction, and clinical complexity. Formal incremental prognostic analyses (C-statistics, NRI, IDI) with direct comparisons with validated risk scores were not performed. Second, this is a single-center study; therefore, extrapolation of the findings to other healthcare systems or levels of care should be undertaken with caution. In addition, the definition of sCRMS is based on simple clinical and laboratory parameters obtained at admission, which, while enhancing applicability, may not fully capture the pathophysiological and biological complexity of the syndrome or its dynamic nature during hospitalization and follow-up. Future prospective studies incorporating continuous variables, body composition measures, and more granular metabolic characterisation are needed to refine the metabolic dimension of severe CRMS. Finally, the long inclusion period may have introduced some heterogeneity related to temporal changes in therapeutic strategies and standards of HF management.

Nevertheless, these limitations are counterbalanced by relevant strengths that underscore the robustness of the results. Notably, the large sample size, with consecutive inclusion over a prolonged period, minimizes selection bias and provides a realistic picture of routine clinical practice. Furthermore, the prospective and structured collection of clinical data by a team specialized in HF ensures high data quality and contributes to the internal validity of the study. From a conceptual standpoint, the integrative sCRMS approach goes beyond the isolated analysis of comorbidities and aligns with current cardiorenometabolic disease paradigms, offering a syndromic perspective that more closely reflects clinical reality. Moreover, the use of a simple, reproducible, and admission-applicable definition facilitates immediate translation into practice, enabling early identification of patients at higher risk.

In summary, in the analyzed cohort, the presence of sCRMS was associated with a high-risk clinical profile with a clear increase in morbidity and mortality. This finding suggests that sCRMS does not act merely as an indirect marker of disease severity or comorbidity burden but rather identifies a phenotype with inherent systemic vulnerability. The results support the usefulness of integrating renal and metabolic variables into prognostic stratification in acute HF. Early identification of this phenotype may allow more accurate risk stratification, guide more intensive follow-up strategies, and facilitate the development of integrated therapeutic interventions along the cardiorenometabolic axis, particularly after hospital discharge, when clinical vulnerability remains high.

## 5. Conclusions

In a large contemporary cohort of patients hospitalized for acute HF, the presence of sCRMS consistently identifies a clinically vulnerable phenotype, characterized by older age, a greater burden of cardiovascular and metabolic comorbidities, a more advanced clinical course, and a more complex hemodynamic profile. From a prognostic standpoint, sCRMS is associated with a significantly higher incidence of HF-related mortality, HF readmissions, and the combined endpoint, acting as an independent predictor of worse clinical outcomes.

## Figures and Tables

**Table 1:** Medical history and clinical profile.

	sCRMS Yes (n = 486)	sCRMS No (n = 1742)	*p*-Value	Total (n = 2228)
Age *	77 (12)	73 (19)	0.0001	74 (17)
Male sex	304 (62.6)	1039 (59.6)	0.249	1343 (3)
Underlying heart disease:				
- AF/Flutter	21 (4.3)	158 (9.1)	0.0001	179 (8)
- Hypertension	73 (15)	209 (12)		282 (12.7)
- Ischemic heart disease	237 (48.8)	436 (25)		673 (30.2)
- Non-ischemic DCM	29 (6)	223 (12.8)		252 (11.3)
- Valvular heart disease	97 (20)	510 (29.3)		607 (27.2)
- Other causes	29 (5.9)	206 (11.8)		235 (10.6)
Previous CV surgery	107 (22.2)	377 (21.7)	0.803	484 (21.8)
HT	464 (95.7)	1270 (72.9)	0.0001	1734 (77.9)
Dyslipidemia	401 (82.5)	938 (53.9)	0.0001	1339 (60.1)
Smoking #	218 (44.9)	680 (39.1)	0.012	898 (40.4)
Alcoholism	29 (6)	139 (8)	0.081	168 (7.5)
COPD	77 (15.8)	256 (14.7)	0.289	333 (15)
Sleep apnea	80 (19.6)	160 (11.7)	0.0001	240 (13.5)
Hypothyroidism	44 (9.4)	146 (8.6)	0.319	190 (8.8)
AF	277 (57.1)	1004 (58)	0.755	1281 (57.8)
Stroke	44 (10.5)	143 (9.8)	0.712	187 (10)
Peripheral vascular disease	76 (18.5)	99 (7.2)	0.0001	175 (9.8)
Number of previous hospitalizations ^	2 (2)	1 (1)	0.0001	1 (1)
De novo HF	64 (13.3)	521 (30.2)	0.0001	585 (26.5)
Days of hospitalization	8 (7)	7 (7)	0.015	7 (7)
Baseline NYHA				
- I, II	280 (57.6)	1212 (69.6)	0.0001	1492 (67)
- III, IV	206 (42.4)	530 (30.4)		736 (33)
Hemodynamic pattern:				
- Low output	23 (4.1)	101 (5.8)	0.001	124 (5.6)
- Pulmonary congestión	254 (52.3)	1075 (61.7)		1329 (59.6)
- Mixed congestion @	132 (27.2)	378 (21.7)		510 (22.9)
- Systemic congestion	77 (16.4)	188 (10.8)		265 (11.9)
CRT	52 (10.7)	139 (8)	0.073	191 (8.6)
ICD	79 (16.3)	213 (12.2)	0.028	292 (13.1)
Preserved LVEF	186 (38.3)	708 (40.6)	0.058	894 (40.1)
Normal RVEF	319 (65.6)	1082 (62.1)	0.253	1401 (63)
Dilated right ventricle	165 (34)	610 (35)	0.440	775 (34.8)

* Kolmogorov–Smirnov: 0.0001. Median and interquartile range. Values are expressed as absolute numbers and percentages (in parentheses). # Current smoker or ex-smoker < 10 years. ^ Includes the hospitalization of the study. @ Pulmonary and systemic congestion. Abbreviations: AF: atrial fibrillation; CV: cardiovascular; COPD: chronic obstructive pulmonary disease; CRT: Cardiac resynchronization therapy; DCM: dilated cardiomyopathy; HF: Heart failure; HT: hypertension; ICD: implantable cardioverter-defibrillator; LVEF: left ventricle ejection fraction; NYHA: New York Heart Association; RV: right ventricle; sCRMS: Severe cardiorenometabolic syndrome.

**Table 2:** Prior treatment.

	sCRMS Yes (n = 486)	sCRMS No (n = 1742)	*p*-Value	Total (n = 2228)
ACEI/ARB/ARNI *	245 (50.4)	928 (53.3)	0.257	1173 (52.6)
Beta-blocker *	326 (67.1)	1000 (57.4)	0.0001	1326 (59.5)
MRA *	160 (32.9)	521 (29.9)	0.198	681 (30.6)
Ivabradine *	27 (5.6)	103 (5.9)	0.905	130 (5.8)
Digoxin *	19 (3.9)	118 (6.8)	0.024	137 (6.1)
Loop diuretic *	403 (82.9)	1033 (59.3)	0.0001	1436 (64.5)
Thiazide *	123 (25.3)	300 (17.2)	0.0001	423 (19)
Tolvaptan *	22 (4.5)	30 (1.7)	0.001	52 (2.3)
Acetazolamide *	23 (4.7)	14 (0.8)	0.0001	37 (1.7)
Antiplatelet *	195 (40.1)	481 (27.6)	0.0001	676 (30.3)
Anticoagulant *	267 (54.9)	873 (50.1)	0.071	1140 (51.2)
Nitrates *	71 (14.6)	110 (6.3)	0.0001	181 (8.1)
Oral antidiabetics *	325 (66.9)	441 (25.3)	0.0001	766 (34.4)
SGLT2 inhibitor *	170 (35)	387 (22.2)	0.0001	557 (25)
Potassium binder *	18 (3.7)	3 (0.2)	0.0001	21 (0.9)
Potassium supplement *	41 (8.4)	96 (5.5)	0.018	137 (6.1)
Antiarrhythmics *	89 (18.3)	228 (13.1)	0.011	317 (14.2)
Statins *	370 (76.1)	904 (51.9)	0.0001	1274 (57.2)
Calcium channel blockers *	218 (44.9)	404 (23.2)	0.0001	622 (27.9)
Pulmonary vasodilator *	12 (2.5)	42 (2.4)	0.853	54 (2.4)
Allopurinol *	208 (42.8)	314 (18)	0.0001	522 (23.4)
Vericiguat *	17 (3.5)	21 (1.2)	0.048	38 (1.7)

* Values are expressed as absolute numbers and percentages (in parentheses). Abbreviations: ACEI: angiotensin-converting enzyme inhibitor; ARB: angiotensin II receptor blocker; ARNI: angiotensin receptor-neprilysin inhibitors; MRA: mineralocorticoid receptor antagonist; sCRMS: Severe cardiorenometabolic syndrome; SGLT2: sodium–glucose cotransporter 2.

**Table 3:** Laboratory findings at admission.

	sCRMS Yes (n = 486)	sCRMS No (n = 1742)	*p*-Value	Total (n = 2228)
Urea (mg/dL)	97 (65)	49 (32)	0.0001	55 (44)
Bilirubin (mg/dL)	0.67 (0.55)	0.87 (0.76)	0.0001	0.81 (0.7)
AST (U/L)	22 (14)	25 (18)	0.0001	24 (17)
ALT (U/L)	17 (15)	21 (19)	0.0001	20 (18)
High-sensitivity Troponin T (ng/L)	57.8 (62.2)	40.5 (55.6)	0.0001	45 (62)
NT-proBNP (pg/mL)	10,147 (17,255)	4622 (7156)	0.0001	5465 (8662)
Sodium (mEq/L)	138 (6)	140 (5)	0.0001	139 (5)
Potassium (mEq/L)	4.6 (1)	4.3 (0.8)	0.0001	4.3 (0.8)
Hemoglobin (g/dL)	11.5 (3.2)	12.7 (2.8)	0.0001	12.3 (3)
Hematocrit (%)	36.7 (9)	40.1 (8.8)	0.0001	38.4 (8.8)
Platelets (µL, ÷100)	198 (85)	218 (105)	0.014	213 (104)
Uric acid (mg/dL)	8.4 (3.3)	7.7 (3.1)	0.0001	7.8 (3.2)
TSI (%)	18 (12)	17 (12)	0.636	17 (12)
Ferritin (ng/mL)	195 (324)	152 (230)	0.0001	159 (250)
HbA1c (%)	6.4 (1.3)	5.9 (1)	0.0001	6 (1)
CA125 (U/mL)	60 (71)	49 (189)	0.535	75.4 (142.4)
Cystatin C (mg/L)	2.12 (1.33)	1.43 (1.2)	0.056	2.07 (1.4)
Urinary creatinine (mg/dL)	43.5 (29.2)	52.4 (58.2)	0.207	50.8 (42.9)
Microalbuminuria (mg/g)	2 (7.4)	12.7 (12.5)	0.975	2.65 (10.78

Kolmogorov–Smirnov: 0.0001. Median and interquartile range. Abbreviations: ALT: alanine aminotransferase; AST: aspartate aminotransferase; CA125: Carbohydrate antigen 125; HbA1c: glycated hemoglobin; NT-proBNP: N-terminal pro–B-type natriuretic peptide; sCRMS: Severe cardiorenometabolic syndrome; TSI: transferrin saturation index.

**Table 4:** Diagnostic and study criteria.

	sCRMS Yes (n = 486)	sCRMS No (n = 1742)	*p*-Value	Total (n = 2228)
Creatinine (mg/dL) #	2.04 (1.01)	1.1 (0.52)	0.0001	1.24 (0.83)
GFR (mL/min/1.73 m^2^) #	30.65 (16.38)	64.5 (36.5)	0.0001	54.85 (42.28)
GFR < 45 mL/min/1.73 m^2^	486 (100)	349 (20)	0.0001	835 (37.5)
Diabetes mellitus	448 (92.2)	565 (32.4)	0.0001	1013 (45.5)
Obesity	116 (23.9)	257 (14.8)	0.0001	373 (16.7)
HF mortality	143 (29.4)	320 (18.4)	0.0001	463 (20.8)
HF readmission	273 (56.2)	584 (33.5)	0.0001	857 (38.5)
HF mortality + readmission	416 (85.6)	904 (51.9)	0.0001	1320 (59.2)
Follow-up Time #	116.5 (356)	111 (325)	0.186	110 (332)

# Kolmogorov–Smirnov: 0.0001. Median and interquartile range. Values are expressed as absolute numbers and percentages (in parentheses). Abbreviations: GFR: glomerular filtration rate; HF: heart failure; sCRMS: Severe cardiorenometabolic syndrome.

**Table 5:** Ten-Year Mortality and Heart Failure Readmissions According to Severe Cardiorenometabolic Syndrome and Its Individual Components.

Phenotype	Total (n)	Deaths (n)	Mortality (%)	*p*-Value vs. Severe CRMS	HF Readmissions (n)	Readmissions (%)	*p*-Value vs. Severe CRMS
Severe CRMS	486	143	29.4	—	273	56.2	—
Isolated Renal Dysfunction (eGFR < 45 mL/min/1.73 m^2^)	835	252	30.2	0.772	429	51.4	0.092
Diabetes Mellitus Alone	1013	247	24.4	0.037	449	44.3	<0.001
Obesity Alone	373	44	11.8	<0.001	159	42.6	<0.001

Abbreviations: CRMS, cardiorenometabolic syndrome; HF, heart failure; eGFR, estimated glomerular filtration rate.

**Table 6:** Event analysis.

sCRMS Phenotype	Unadjusted			Adjusted ^&^		
	HR	95%CI	*p*	HR	95%CI	*p*
Mortality	1.558	1.278–1.898	0.0001	1.252	1.016–1.544	0.035
HF Readmissions	2.541	2.070–3.119	0.0001	1.235	1.061–1.438	0.006
Mortality + Readmissions	1.516	1.328–1.730	0.0001	1.201	1.045–1.381	0.01

^&^: Adjusted for age, sex, baseline NYHA class, de novo heart failure, ischemic etiology, and variables clinically relevant or significant in univariable analysis. They were also significant for mortality: Age (HR: 1.031, 95% CI: 1.022–1.040) and de novo HF (HR: 0.625, 95% CI: 0.474–0.826). For readmission: Age (HR: 1.014, 95% CI: 1.007–1.020), baseline functional class I–II vs. III–IV (HR: 0.501, 95% CI: 0.259–0.972), and de novo HF (HR: 0.049, 95% CI: 0.026–0.091). For mortality + readmissions: Age (HR: 1.019, 95% CI: 1.013–1.025) and de novo HF (HR: 0.278, 95% CI: 0.217–0.356). Abbreviations: CI: confidence interval; HF: heart failure; sCRMS: Severe cardiorenometabolic syndrome.

## Data Availability

The dataset is available on request from the authors.
